# Obestatin controls skeletal muscle fiber-type determination

**DOI:** 10.1038/s41598-017-02337-4

**Published:** 2017-05-18

**Authors:** Icía Santos-Zas, Tania Cid-Díaz, Jessica González-Sánchez, Uxía Gurriarán-Rodriguez, Carlos Seoane-Mosteiro, Begoña Porteiro, Rubén Nogueiras, Xesús Casabiell, José Luis Relova, Rosalía Gallego, Vincent Mouly, Yolanda Pazos, Jesus P. Camiña

**Affiliations:** 1 Área de Endocrinología Celular y Molecular, Instituto de Investigación Sanitaria de Santiago (IDIS), Complejo Hospitalario Universitario de Santiago (CHUS), Servicio Gallego de Salud (SERGAS), Santiago de Compostela, Spain; 2 Sprott Center for Stem Cell Research, Ottawa Hospital Research Institute, Ottawa, ON K1H8L6 Canada; 3 Departamento de Fisiología, Universidad de Santiago de Compostela (USC), Santiago de Compostela, Spain; 4 Departamento de Ciencias Morfológicas, USC, Santiago de Compostela, Spain; 5 Sorbonne Universités, UPMC Univ Paris 06, INSERM UMRS974, CNRS FRE3617, Center for Research in Myology, Paris, France

## Abstract

Obestatin/GPR39 signaling stimulates skeletal muscle growth and repair by inducing both G-protein-dependent and -independent mechanisms linking the activated GPR39 receptor with distinct sets of accessory and effector proteins. In this work, we describe a new level of activity where obestatin signaling plays a role in the formation, contractile properties and metabolic profile of skeletal muscle through determination of oxidative fiber type. Our data indicate that obestatin regulates Mef2 activity and PGC-1α expression. Both mechanisms result in a shift in muscle metabolism and function. The increase in Mef2 and PGC-1α signaling activates oxidative capacity, whereas Akt/mTOR signaling positively regulates myofiber growth. Taken together, these data indicate that the obestatin signaling acts on muscle fiber-type program in skeletal muscle.

## Introduction

The heterogeneous population of muscle fibers determines the plasticity of skeletal muscle^1^. Slow-twitch (type I) myofibers are rich in mitochondria, have high oxidative capacity and capillary density, whereas fast-twitch (type II) fibers have lower mitochondrial and capillary density and generate ATP primarily through glycolysis^1^. In addition, the structural and functional properties of the fibers can undergo conversion between them in response to exercise training, motor neuron activity or hormonal/growth factor signaling^2^. Among the growth factors that play a role in muscle plasticity, the insulin-like growth factor-I (IGF-I and mechano growth factor)^3,4^ and the transforming growth factor-β superfamily member, myostatin^5,6^ are described as key regulatory factors. However, skeletal muscle is also a secretory organ^7^, and this concept highlights the importance of secreted molecules not only in orchestrating muscle regeneration but also in mediating the plasticity of muscle. Specific perturbations of the secreting activity during pathological processes such as dystrophic conditions further highlight the importance of these secreted molecules^8^.

Obestatin, a 23-amino acid peptide derived from a polypeptide called preproghrelin, exerts an autocrine function through the G-protein-coupled receptor GPR39 to control the myogenic differentiation program^9^. Obestatin is expressed in healthy skeletal muscle, and this expression is strikingly increased upon muscle injury. Obestatin is coordinately up-regulated during the early stages of myogenesis, and its level remains sustained throughout terminal differentiation^9^. The obestatin/GPR39 system up-regulates the satellite cells marker Pax7, myogenic factors and embryonic myosin heavy chain (eMHC). This action is associated to the regulation on the different stages of myogenesis: proliferation, migration, fusion and myofiber growth, and more generally enhancing the regeneration process with the formation of fibers of larger caliber in mouse skeletal muscle^10^. In a human context *in vitro*, the obestatin/GPR39 system exerts an autocrine effect on the skeletal myogenic process promoting myogenic differentiation and fusion of human myoblasts^11^. At the molecular level, the action of obestatin involves both G-protein-dependent and -independent mechanisms linking the activated GPR39 receptor with distinct sets of accessory and effector proteins, thereby controlling the specificity and efficiency of the signals. The obestatin-associated mitogenic action is determined by G-protein-dependent activation defining the intricate pathways related to the ERK1/2 and JunD axis. The transactivation of EGFR through the β-arrestin signal complex determines the cell cycle exit, the development and the progression of obestatin-dependent differentiation through a kinase hierarchy determined by the Akt, CAMKII, c-Jun and p38 axes. The cross-talk involves the functional interaction between β-arrestins and GPR39 leading to Src activation and signalplex formation to EGFR transactivation by matrix metalloproteinases (MMPs). Thus, ß-arrestins arbitrate cell fate between G-protein-dependent and β-arrestin-dependent signaling pathways to direct the progression of the myogenic program. This dual mechanism highlights the importance of β-arrestins as key specific regulator of the progression within the myogenic program, by triggering intracellular activity patterns. Taken together, these data elucidate a mechanism whereby obestatin and GPR39 is coordinately regulated as part of the myogenic program and operates as an autocrine signal regulating skeletal myogenesis.

Obestatin was originally identified in the stomach as a physiological opponent of ghrelin^12^. However, this peptide is not free from controversy. The first outstanding fact was its opposite function to ghrelin orexigenic action. The second, but not less critical, was its receptor, GPR39^13,14^. Very soon after Zhang’s work was published, several groups discarded its actions regarding feeding^15–19^. Only one year later, Holst B. *et al*. published their work regarding the GPR39 receptor: obestatin was not able to activate this receptor, but Zn^2+^ did^14^. However, all these results might be questionable. It was not until 2008 that De Spiegleer B *et al*. demonstrated the quality of the obestatin peptides supplied by several companies concluding that these peptides were not suitable to obtain good and unquestionable results^20^. Regarding the obestatin binding assays to GPR39, the same group demonstrated in 2012 that iodinated obestatin was also a mixture of poly-iodinated peptides^21^. Indeed, already in 2008, Zhang J *et al*. mentioned this subject^22^. Amid all this controversy, it was further reported that obestatin biological effect is species-specific: mouse and human obestatin have different primary and secondary structures and, in consequence, different activities^23^. All these facts might explain all controversial arising around obestatin action. Despite this controversy, there had some noteworthy results regarding obestatin bioactivity along these years. This peptide displays a variety of cellular effects, by regulating metabolic and cell differentiation functions, increasing cell survival and proliferation, and inhibiting apoptosis and inflammation in different cell types^24^. In particular, obestatin regulates adipogenesis^25^, pancreatic homeostasis^26^ and is involved in gastric cancer^27^. In adipogenesis, for example, the obestatin/GPR39 system exerts a regulatory role on the expression of master regulators of the adipocyte fate and, consequently, lipid accumulation^25^. The relevance of obestatin as a regulator of adipocyte metabolism was further supported by GLUT4 translocation, and increased glucose uptake^25^ being a mediator for insulin-induced adipogenesis^28^.

Several groups have designated Zn^+2^ as a ligand for GPR39^14^ and some others considered that GPR39 is the Zn^+2^-sensing receptor (ZnR). They reported that GPR39, activated by Zn^+2^, has a role in promoting proliferation^29^ and enhanced survival of colonocytes^30^ voltage and cancer prostate cells^31^. However, Zn^+2^ has also been described as an apoptotic agent in several human cancer cells: melanoma^32^, glioma^33^, bladder^34^, prostate^35^ and breast cancer cells^36^. Treatment with Zn^+2^ triggers the activation of MAPK and PI3K/Akt signaling pathways through EGFR activation in several cell types^37–39^. These findings also suggest that there are specific marked differences to each cell type in the EGFR activation mechanism induced by Zn^+2 ^^38^. These contradictory results added more questions to the intriguing relationship between Zn^+2^ and the GPR39 receptor. Additionally, Zn^+2^ treatment was described to diminish GPR39 basal phosphorylation^40^. Having all this information in mind, these data could imply Zn^+2^ in GPR39 signaling, probably due to the activation of the MMPs in EGFR pathways, since obestatin needs EGFR transactivation and MMPs activity^11,27^. Indeed, Zn^+2^ induces EGFR phosphorylation through the extracellular release of EGF-like ligands that are mediated by MMPs^39,41^. On the other hand, it was reported that obestatin action might be mediated partially by the glucagon-like peptide-1 (GLP-1) receptor (GLP1R) in pancreatic β-cells, which would explain its insulinotropic and survival effects^26^. However, this was questioned based on results obtained from INS-1β and HEK cells overexpressing GLP1R in which obestatin was not able to displace radiolabelled GLP-1 binding^42^ and the fact that this work is prior to the report about quality of obestatin peptides. Furthermore, to the best of our knowledge, there is no data supporting the role of GLP-1R, i.e. siRNA technology, on obestatin signaling unlike GPR39^9–11^. First, knockdown of GPR39 by siRNA significantly inhibited obestatin signaling in murine and human myoblasts^9,11^. This is further supported by siRNA assays developed in murine adipocytes^25^, human gastric adenocarcinoma^43^ and retinal-pigmented epithelium cells^23^. Second, coimmunoprecipitation experiments demonstrated the binding of obestatin to GPR39 in cultured C2C12 myoblast cells^10^. These data not only demonstrate the implication of GPR39 in obestatin signaling but also reveals the complexity of this system.

The capacity of obestatin signaling to enhance muscle fiber formation led us to investigate whether it could also act on muscle fiber-type program in skeletal muscle. We found that, in addition to the well-established role in the control of muscle regeneration, obestatin participates in the specification of muscle fiber identity by inducing skeletal muscle remodeling toward an oxidative phenotype. This shift in fiber type results in enhanced muscle strength. Obestatin acts through both class II histone deacetylases (HDAC)/myocyte enhancer factor-2 (Mef2) and peroxisome proliferator-activated receptor-gamma coactivator 1 (PGC-1) mechanisms, thereby controlling the establishment of oxidative muscle fibers. *In vitro* studies indicate that obestatin can induce a shift in fiber type during human myogenesis. Taken together, these data indicate that obestatin signaling regulates muscle fiber type and metabolism.

## Results

### Dose-response effects of obestatin administration on recovery of muscle function

**Figure 1:**
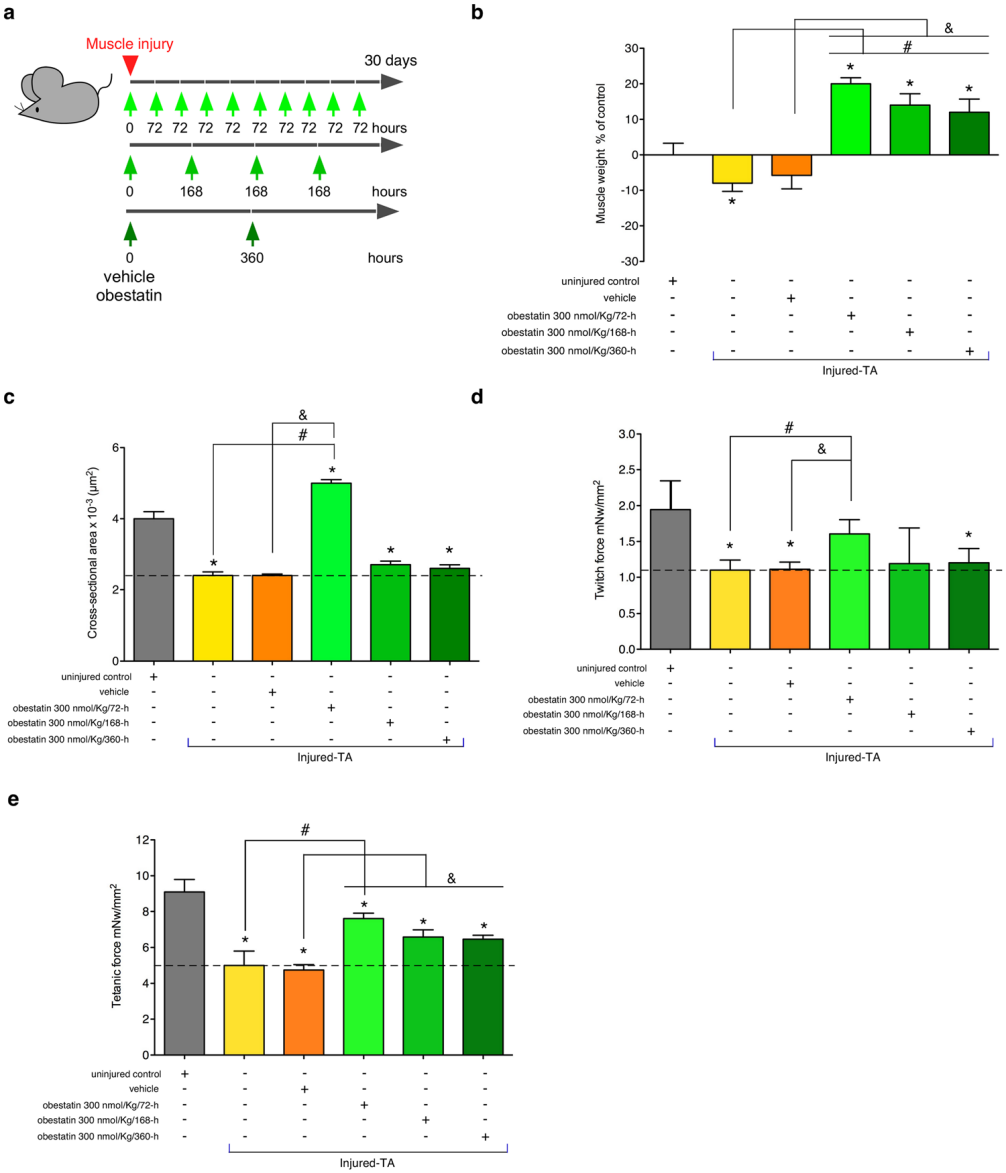
Effect of the timing of intramuscular administration of obestatin following muscle injury. (**a**) A schematic diagram of the muscle injury and regeneration experiment. (**b**) Tibialis anterior (TA) muscle weight after 30 days of treatment with vehicle or obestatin at 300 nmol/kg body weight each 72-, 168- or 360-h. Muscle weights were normalized by body weights and expressed as percent changes from uninjured control. Data are shown as mean ± SEM of 5 animals per group (*^,#,&^P < 0.05 *versus* control values). (**c**) Cross sectional area of muscle fibers from TA muscles after intramuscular injection of obestatin [300 nmol/kg body weight each 72-, 168 or 360-h; n = 5 per group] at 30 days. Data are shown as mean ± SEM of 5 animals per group (*^,#,&^P < 0.05 *versus* control values). (**d**) Effect of intramuscular injection of obestatin [300 nmol/kg body weight each 72-, 168 or 360-h; n = 5 per group] on twitch force at 30 days following injury (*^,#,&^P < 0.05 *versus* control values). (**e**) Effect of intramuscular injection of obestatin [300 nmol/kg body weight each 72-, 168 or 360-h; n = 5 per group] on tetanic force at 30 days following injury. Data in **c** and **d** are expressed as mean ± SEM (*^,#,&^P < 0.05 *versus* control values).

Previous results demonstrated that obestatin induced repair and growth by stimulating both satellite cell expansion and myofiber hypertrophy. However, whether this resulted in an increased specific force remains unanswered. To address this question, the force generated by TA muscles from obestatin-treated mice was measured. In the first part of this study, the 300 nmol/Kg body-weight dose of obestatin was used for the timing studies^10^. Obestatin or vehicle (PBS) was directly injected into the TA muscles of 10-weeks-old mice (n = 5/group) following freeze injury with three timing of administration, every 72-, 168- or 360-h during 30 days (Fig. 1a). Obestatin treatment led to a significant ~14–12% increase in muscle weight of TA treated each 168- or 360-h in relation to uninjured control muscles (Fig. 1b). The increase in muscle weight was more marked in TA treated each 72-h with a ~20% increase in muscle weight. The histological analysis revealed that the treated-to-control ratios of cross-section area was ~25% larger in obestatin-treated mice compared to uninjured control mice 30-days after injury (Fig. 1c). The increase was even more enhanced when compared to vehicle treated-muscles with a ~108% increase in cross-section area (Fig. 1c). The increase in fiber size and muscle mass in obestatin-treated muscle was concomitant with significant increases in the specific force (force normalized to muscle fiber cross-sectional area) generated by TA muscles, as compared to injured-muscles. Mean twitch and tetanic force of normal TA were respectively 1.95 and 9.04 mN/mm^2^ (Fig. 1d and e, respectively). Mean twitch and tetanic force were decreased 30 days after the induction of injury as the means were 1.10 and 5.0 mN/mm^2^ (~44 and 45% loss of force), or 1.11 and 4.56 mN/mm^2^ after vehicle administration (44 and 50% loss of force), respectively. By contrast, mean twitch and tetanic specific force was higher in obestatin-treated TA (300 nmol/Kg/72-h) as the means were 1.61 and 7.61 mN/mm^2^, representing a 17 and 16% loss of force regarding normal TA, though 45 and 67% recovery of force regarding vehicle-treated TA (Fig. 1d and e, respectively).

**Figure 2:**
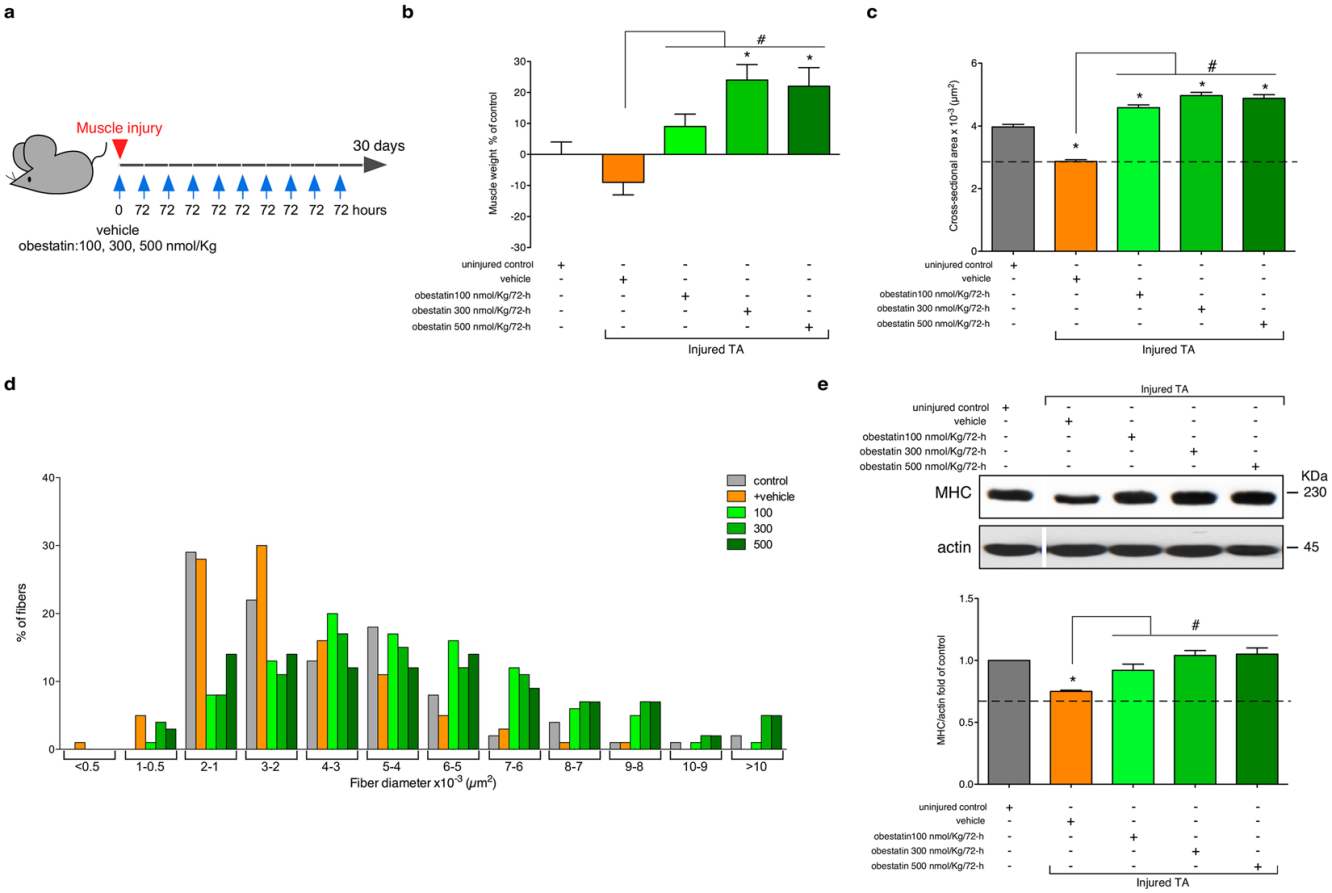
Obestatin treatment increases muscle mass and fiber diameter. (**a**) A schematic diagram of the muscle injury and regeneration experiment. (**b**) Tibialis anterior (TA) muscle weight after 30 days of treatment with vehicle or obestatin at 100-, 300- or 500-nmol/kg/72-h. Muscle weights were normalized by body weights and expressed as percent changes from uninjured control. Data are shown as mean ± SEM of 5 animals per group (*^,#^*p* < 0.05 *versus* control values). (**c**) Cross sectional area of muscle fibers from TA muscles after intramuscular injection of obestatin [100-, 300- or 500-nmol/kg/72-h; n = 6 per group] at 30 days. Data are shown as mean ± SEM of 5 animals per group (*^,#^*p* < 0.05 *versus* control values). (**d**) Distribution of fiber diameter from control, vehicle- and obestatin-treated mice. (**e**) Immunoblot analysis of the expression of MHC in uninjured control and freeze-injured TA muscles after intramuscular injection of obestatin (100-, 300- or 500-nmol/kg/72-h; n = 6 per time point) or vehicle (PBS) at 30 days following injury. Protein levels were expressed as fold of control (n = 6/group). Dividing lines (white lines) indicate splicing of the same gel. Full-length blots are presented in Supplementary Figure 1. Data were expressed as mean ± SEM obtained from intensity scans (*^,#^*p* < 0.05 *versus* control values).

**Figure 3:**
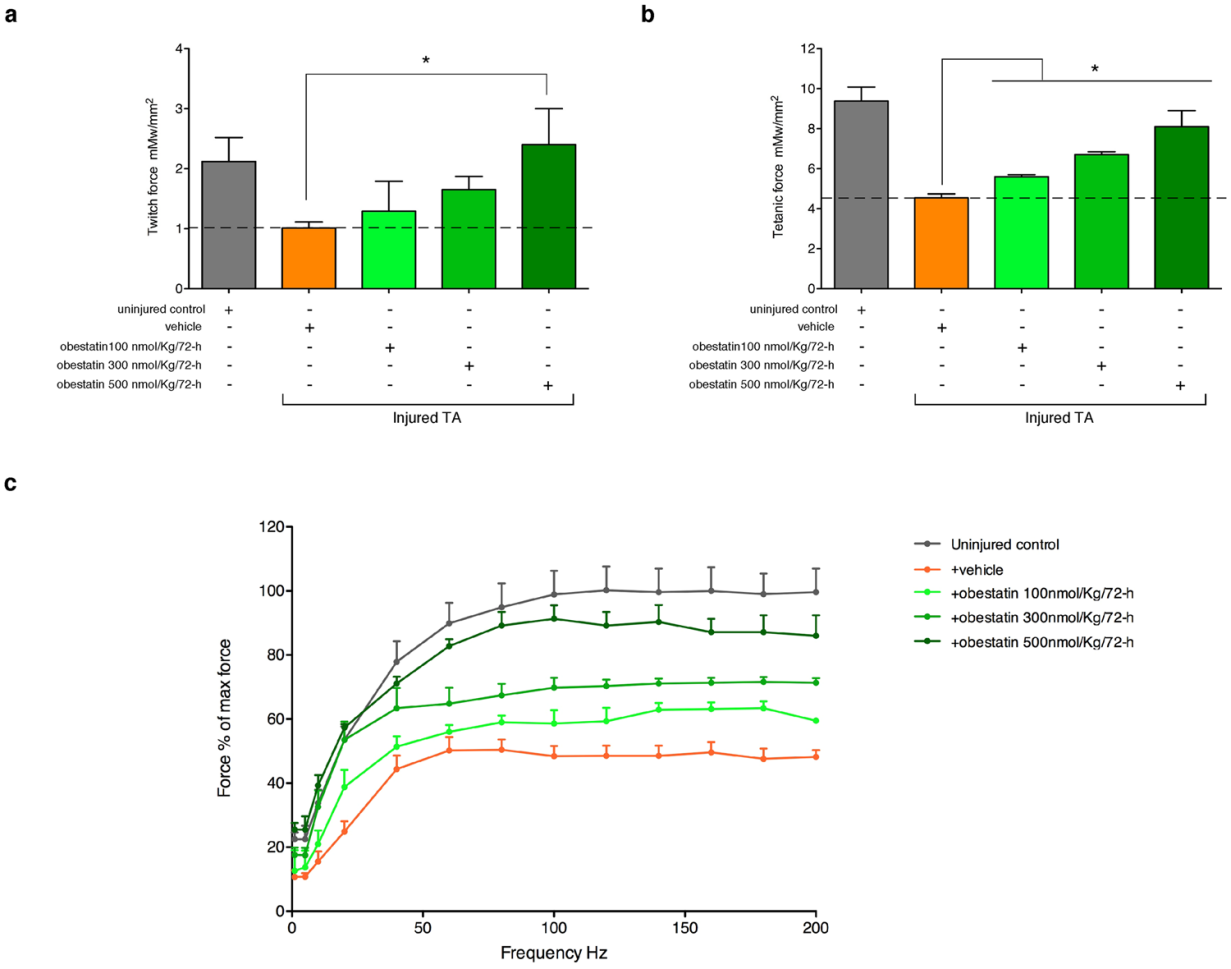
Obestatin increases muscle force. Obestatin or PBS (vehicle) was administrated via intramuscular injection in freeze-injured TA muscle (100-, 300 or 500-nmol/kg/72-h during 30 days). Uninjured TA muscles were taken as control to establish that the damage area was large enough to decrease force (n = 6 per group). Muscle force measurements were assessed after the last dose. (**a**) Effect of intramuscular injection of obestatin on twitch force at 30 days following injury. (**b**) Effect of intramuscular injection of obestatin on tetanic force at 30 days following injury. (**c**) Force-frequency curve of TA muscles in control and obestatin- or PBS-treated groups. Data in **a** and **b** were expressed as mean ± SEM (*p < 0.05 *versus* injured muscle+vehicle control values). Data in **c** were expressed as a percentage of the maximal force generated at 200 Hz (mean ± SEM).

The second part of the study, the optimal dose of obestatin was evaluated (Fig. 2a). The 100, 300 or 500 nmol/Kg body-weight dose of obestatin was used for the dosing studies. Obestatin or vehicle (PBS), initiated immediately after injury, was directly injected into the TA muscles of 10-weeks-old mice (n = 6/group) every 72-h during 30 days. As observed above, no significant differences in muscle weight, cross sectional area and twitch/tetanic force were found between the injured-control and injured-control muscles plus vehicle muscles (vehicle-treated TA muscles; data not shown). For a simplified data analysis, obestatin-treated groups were compared to uninjured control and vehicle-treated TA muscles. Obestatin administration resulted in increased muscle mass of TA that reached maximal effect at 300 nmol/Kg each 72-h, representing a ~24 or 33% increase of wet weight in relation to uninjured control or vehicle-treated TA muscles (Fig. 2b). As shown in Fig. 2c, the treated-to-control ratios of fiber cross-section areas, were dose dependent and increased at maximal levels at 300 nmol/Kg/72-h, above which no additional effect was observed. At 30-days post-injury, cross-section area analysis revealed a significant increase, as compared to uninjured control, in the percentage of myofibers of larger area (Fig. 2d). Furthermore, the protein levels of myosin heavy chain (MHC), as detected by immunoblot, were up-regulated at a maximal level after treatment at 300 nmol/Kg/72-h (~39% increase compared to vehicle-treated TA muscles; Fig. 2e). Despite histological and molecular examination of standard myogenic markers for efficient regeneration reached maximal values at 300 nmol/Kg/72-h, maximum force production capability of TA was obtained at 500 nmol/Kg/72-h. As Fig. 3a and b shows, mean twitch and tetanic force of normal TA were respectively 2.11 and 9.3 mN/mm^2^. Mean twitch and tetanic force were lower in vehicle-treated injured TA as the means were 1.01 and 4.53 mN/mm^2^ (~52 and 51% loss of force), respectively. In obestatin-treated TA, mean twitch and tetanic force were 1.29 and 5.60, 1.65 and 6.7, 2.40 and 8.10 mN/mm^2^ at 100, 300 and 500 nmol/Kg/72-h, respectively. This effect represented a 137% and 78% increase of twitch and tetanic force for muscles treated with 500 nmol/Kg/72-h obestatin, as compared to vehicle-treated TA. These results demonstrate that obestatin not only enhances regenerative capacity, but also leads to increased force generated by the regenerated and treated muscle.

### Obestatin regulates oxidative fiber expression

**Figure 4:**
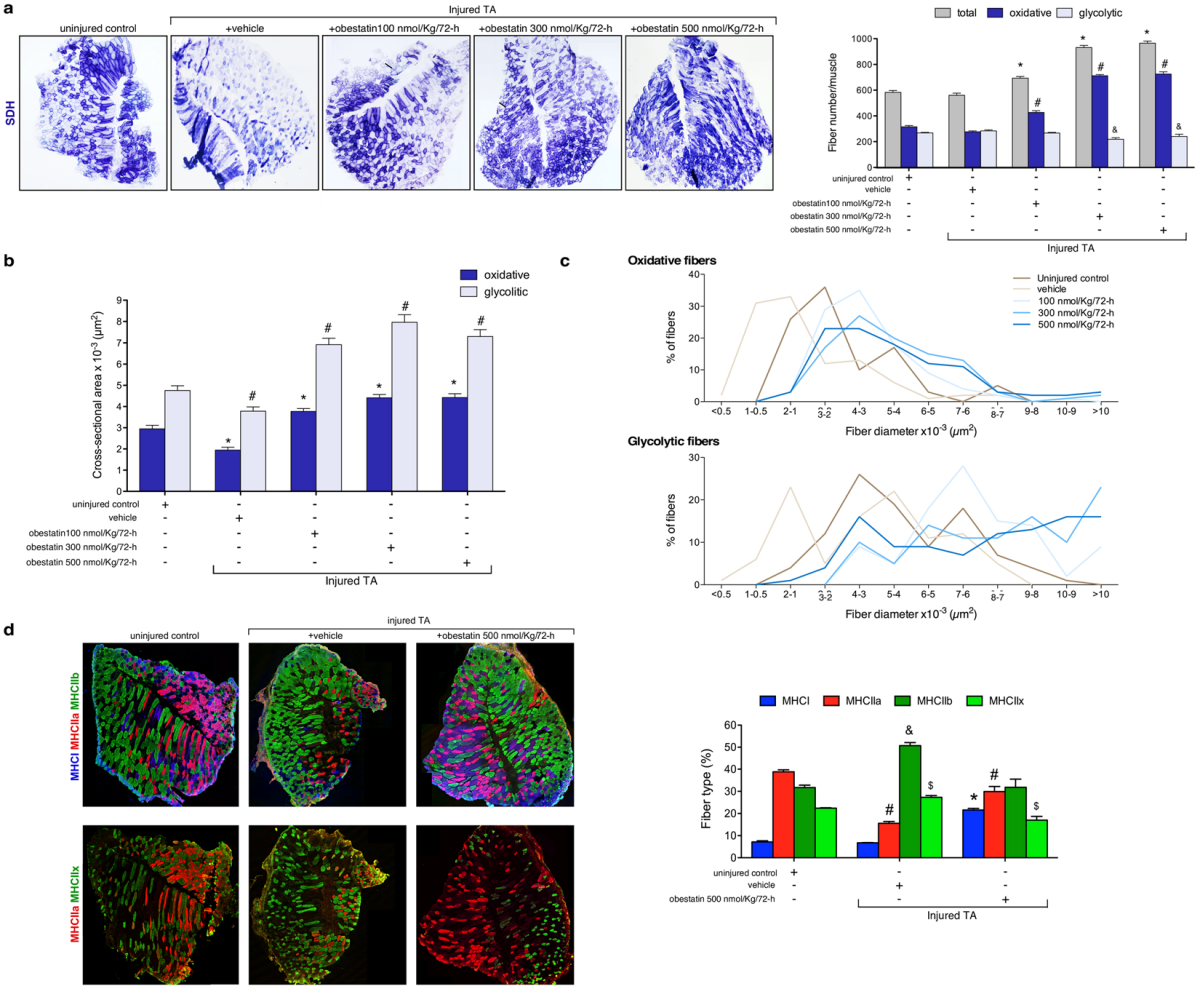
Obestatin increases oxidative fiber density and fiber diameter. (**a**) Left panel, succinate dehydrogenase (SDH) staining from TA from uninjured control, vehicle- and obestatin-treated muscles. Right panel, quantitation of total, glycolytic and oxidative muscle fibers from TA muscles (100-, 300 or 500-nmol/kg/72-h during 30 days). Data are shown as mean ± SEM 6 animals per group (*^,#,&^*p* < 0.05 *versus* control values). (**b**) Cross sectional area of glycolytic and oxidative muscle fibers from TA muscles after intramuscular injection of obestatin. Data are shown as mean ± SEM of 6 animals per group (*^,#^*p* < 0.05 *versus* control values). (**c**) Distribution of oxidative and glycolytic fiber diameters from control, vehicle- and obestatin-treated mice. **(d**) Left panel, representative images of uninjured control, vehicle- and obestatin-treated TA muscles showing MHC expression. Mouse muscle serial cross-section incubated with a primary antibody cocktail against MHCI, MHCIIa, and MHCIIb (upper panels) or MHCIIa, and MHCIIx (lower panels), followed by incubation with appropriate fluorescent-conjugated secondary antibodies. Right panel, quantitation of fiber types. Data are shown as mean ± SEM of 6 animals per group (^*,#,&,$^*p* < 0.05 *versus* control values).

**Figure 5:**
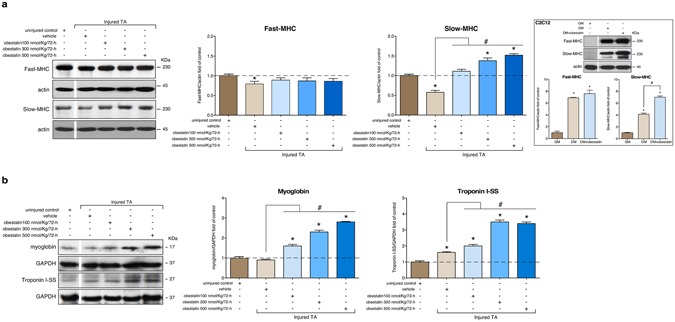
Obestatin stimulation is sufficient to increase slow fiber expression in skeletal muscle. (**a**) Western blot analysis of slow and fast MHC expression in uninjured control and freeze-injured TA muscles after intramuscular injection of obestatin (100-, 300- or 500-nmol/kg/72-h; n = 6 per time point) or vehicle (PBS) at 30 days following injury. The inserted figure shows Western blot analysis of slow and fast MHC expression in C2C12 myoblasts (GM) and myotubes obtained under DM or DM+obestatin (10 nM, 7 days). (**b**) Expression of the slow-fiber–specific troponin I-SS and oxidative marker myoglobin in uninjured control and freeze-injured TA muscles. In **a** and **b** protein level was expressed as fold of uninjured control TA muscles. In **a** and **b** immunoblots are representative of the mean value. In **a** and **b**, dividing lines (white lines) indicate splicing of the same gel. Full-length blots are presented in Supplementary Figure 2. Data were expressed as mean ± SEM obtained from intensity scans (*^,#^*p* < 0.05 *versus* control values).

When specific force-frequency curves were generated, obestatin treatment resulted in a leftward dose-dependent shift in the force-frequency (Fig. 3c). This shift of the curve may result from an increase in force, but also from a shift in fiber types toward an increased amount of slow-twitch fibers. On histological examination of TA (fast/glycolytic muscle) from obestatin-treated animals, there was a marked dose-dependent increase in the relative density of oxidative, SDH positive fibers, compared with controls (uninjured- and vehicle-treated TA), with a corresponding decrease in glycolytic, SDH negative fibers (Fig. 4a). Quantitation of the total fiber number revealed an increase of 19, 60 and 65% in obestatin-treated TA compared to uninjured control at 100-, 300- and 500-nmol/Kg/72-h, respectively (Fig. 4a). This rise was due to a specific dose-dependent increase in the number of oxidative fibers of 36, 126 and 130% compared to uninjured control at 100-, 300- and 500-nmol/Kg/72-h, respectively. On the other hand, the number of glycolytic fibers was decreased by 10 and 18% in obestatin-treated muscles at 300- and 500-nmol/Kg/72-h. Interestingly, despite the reduction in the number of glycolytic fibers, the cross-sectional area of the remaining glycolytic fibers was significantly increased by 45–67% in TA. Cross-sectional area of oxidative fibers was also increased by 28–50% in TA (Fig. 4b). These changes in fiber size were associated with a change and broadening of distribution for both the glycolytic and oxidative fibers, as reflected in Fig. 4c. The slow type I and the fast type IIa, IIb and IIx constitute the four basic fiber types, classically identified based on the molecular properties of their MHC isoforms^44,45^. Immunofluorescence analysis on single muscle cross-section was able to identify the four major fiber types, notably type I (blue), type IIa (red), type IIb (green), and type IIx (unstained; Fig. 4d, upper panel). In addition, the identity of these unstained fibers as type IIx was confirmed by staining serial cross-sections using an antibody specific for MHCIIx (fibers positive for MHCIIx showed green staining; Fig. 4d, lower panel). Muscle fiber-type assessment revealed that obestatin-treated muscles at 500-nmol/Kg/72-h led to the appearance of considerable numbers of type I fibers in TA compared with uninjured control mice (2.9-fold increase; Fig. 4d). Furthermore, these TA muscles had a reduction in type IIa and IIx fiber densities compared with uninjured controls while no significant differences were observed in type IIb fiber density (Fig. 4d). Western blot analysis of fast- and slow-MHC demonstrated a robust increase in the protein levels of slow-MHC, which was up-regulated at a maximal level in the presence of 500 nmol/Kg/72-h obestatin (52% compared to uninjured control) with no significant change in total levels of fast-MHC (Fig. 5a). This was further confirmed *in vitro* in C2C12 cells, which were switched to DM supplemented with 5 nM obestatin for 7 days^10^ showing a significant increase in slow-MHC compared with differentiated control cells (DM; Fig. 5a, inserting image). In addition, there was an increase in the expression levels of genes related to structural proteins of the type I fiber (slow twitch oxidative, red muscle), such as, troponin I slow type (troponin I-SS; 100–240% increase compared to uninjured control TA) and myoglobin (60–180% increase compared to uninjured control TA), which are more abundant in type I fibers (Fig. 5b). Thus, we conclude that obestatin can control fiber type determination, favoring the formation of oxidative slow-twitch fibers after regeneration.

### Signaling pathways involved in slow-twitch-fiber gene expression

**Figure 6:**
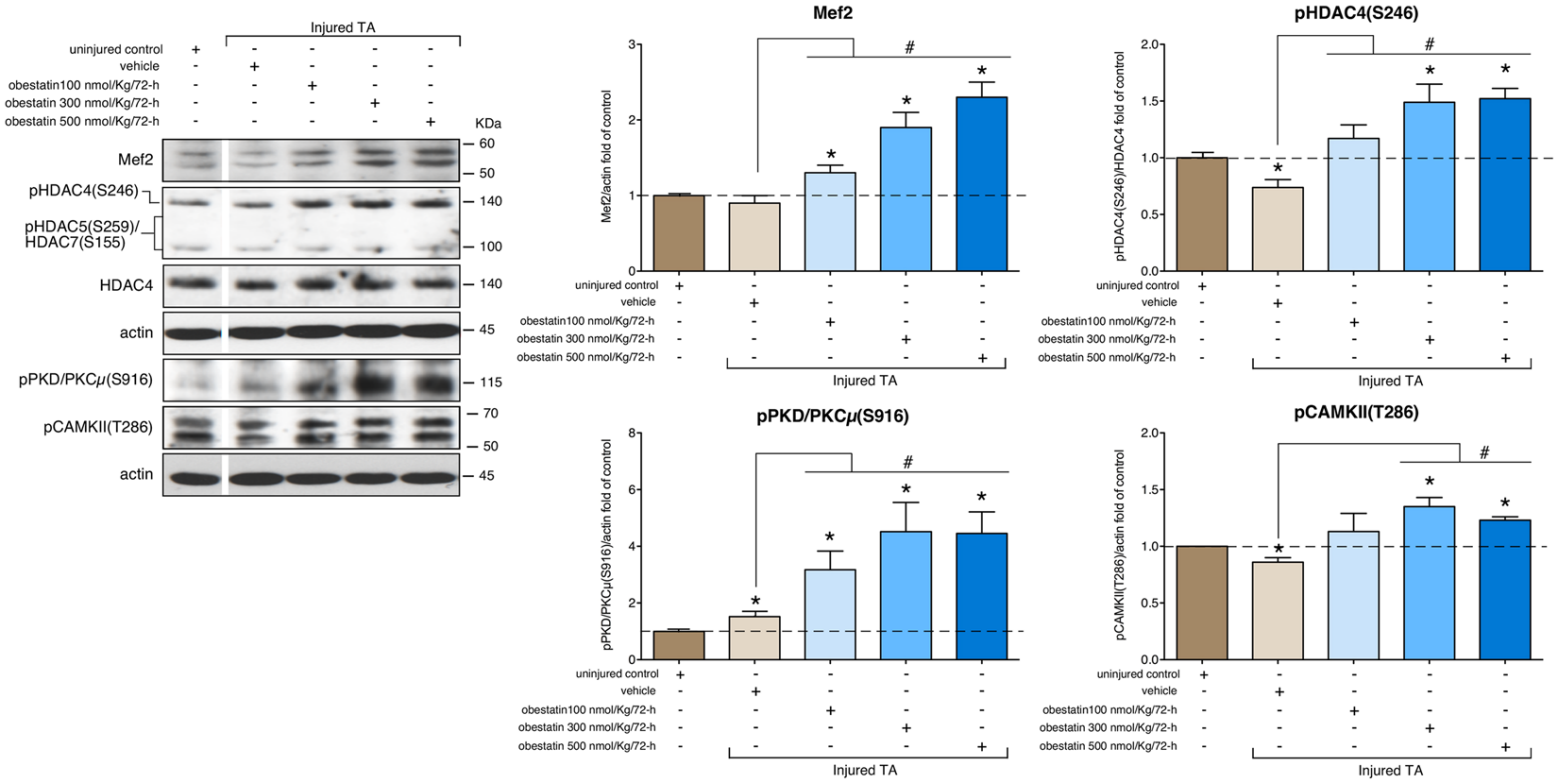
Obestatin signaling regulates HDAC phosphorylation related to slow fiber expression in skeletal muscle. Western blot analysis of Mef2, pHDAC(S246), pHDAC5(S259), pHDAC7(S155), HDAC4, pPKD/PKCμ(S916) and pCAMKII(T286) in uninjured control and freeze-injured TA muscles after intramuscular injection of obestatin (100-, 300- or 500-nmol/kg/72-h; n = 6 per time point) or vehicle (PBS) at 30 days following injury. Protein level was expressed as fold of uninjured control TA muscles. Immunoblots are representative of the mean value. Dividing lines (white lines) indicate splicing of the same gel. Full-length blots are presented in Supplementary Figure 3. Data were expressed as mean ± SEM obtained from intensity scans (*^,#^*p* < 0.05 *versus* control values).

Slow and oxidative myofiber identity is regulated by the balance between positive and negative signaling by Mef2 and the class II HDAC proteins, respectively^46^. Indeed, western blot analysis of obestatin-treated TA muscles demonstrated a robust increase in Mef2 protein up-regulated at a maximal level in the presence of 500 nmol/Kg/72-h, representing a 130% increase in relation to uninjured control TA (Fig. 6). However, the activity of Mef2 is tightly regulated through association with HDACs, which act as signal-dependent repressors of gene expression^47,48^. In response to differentiation signals or motor innervation, HDACs are phosphorylated, a process which provides docking sites for the 14-3-3 chaperone protein that leads to nuclear export of HDACs enabling Mef2 to activate the slow myofiber gene program^47,49,50^. Consistent with this assumption, the obestatin treatment resulted in markedly elevated levels of phosphorylation of HDAC4 [pHDAC4(S246)] reaching maximal levels at 300 nmol/Kg/72-h (Fig. 6). We did not observe an increased phosphorylation of HDAC5(S259) or HDAC7(S155) in TA muscles. We further investigated the implication of calcium-regulated protein kinases, protein kinase D (PKD) and calcium calmodulin-dependent protein kinase II (CaMKII), responsible for phosphorylation of HDACs on a series of conserved serine residues^50^. The activation of PKD [pPKD/PKCµ(S916)], as detected by immunoblot, was dose-dependent being up-regulated at a maximal level in the presence of 500 nmol/Kg/72-h (350% increase compared to uninjured control TA; Fig. 6). The increase was not so clear concerning the activation of CAMKII, estimated as phosphorylation of CAMKII at T286 [pCAMKII(T286)]. In this particular case, maximal levels were reached at 300 nmol/Kg/72-h, representing a 35% increase in relation to uninjured control TA (Fig. 6). Therefore, the regulated phosphorylation of HDAC4 by PKD and CAMKII provides a mechanism for the modulation of Mef2 target genes in response to obestatin to promote the formation of slow-twitch muscle fibers.

**Figure 7:**
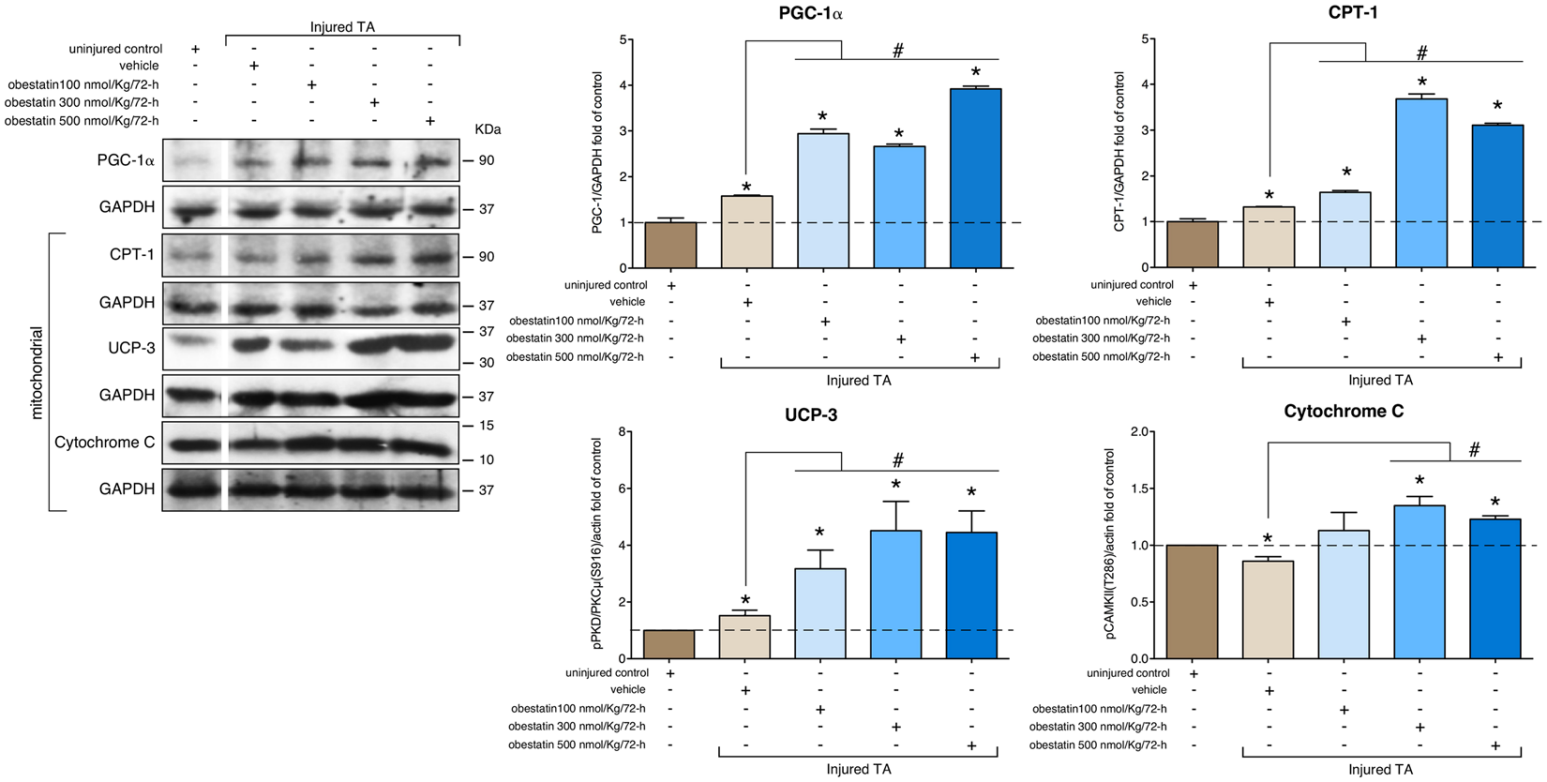
Obestatin signaling regulates the expression of PGC-1α and oxidative markers CPT-1, UCP-3 and Cytochrome C in skeletal muscle. Western blot analysis of PGC-1α, CPT-1, UCP-3 and Cytochrome C in uninjured control and freeze-injured TA muscles after intramuscular injection of obestatin (100-, 300- or 500-nmol/kg/72-h; n = 6 per time point) or vehicle (PBS) at 30 days following injury. Protein level was expressed as fold of uninjured control TA muscles. Immunoblots are representative of the mean value. Dividing lines (white lines) indicate splicing of the same gel. Full-length blots are presented in Supplementary Figure 4s. Data were expressed as mean ± SEM obtained from intensity scans (*^,#^*p* < 0.05 *versus* control values).

In addition to the expression of particular myofibrillar proteins, slow oxidative type I fibers are much higher in mitochondrial content and are more dependent on oxidative metabolism than fast glycolytic type II fibers. We analyzed a transcriptional co-activator, peroxisome-proliferator-activated receptor-γ coactivator-1 (PGC-1α) that activates mitochondrial biogenesis and oxidative metabolism in muscle^51^. The expression of PGC-1α, as detected by western blot, was up-regulated showing maximal level in the presence of 500 nmol/Kg/72-h (292 or 248% increase compared to uninjured control or vehicle-treated TA, respectively; Fig. 7). Immunoblot analyses showed that the expression of mitochondrial proteins was also induced: In TA muscles, expression of Cytochrome C, carnitine palmitoyltransferase-1 (CPT-1), and mitochondrial uncoupling protein 3 (UCP-3) proteins were increased in obestatin-treated mice as compared to uninjured control or vehicle-treated animals (Fig. 7). This up-regulation supports the fiber type conversion toward a more oxidative fiber type during muscle regeneration. Thus, both PGC-1α and Mef2 are involved in the regulation of the expression of slow-twitch myofiber genes and serve as transcriptional targets of the upstream signaling pathways involved in specification of the slow myofiber phenotype under obestatin/GPR39 activation.

### Signaling pathways implicated in the specification of the slow contractile protein gene expression in humans

**Figure 8:**
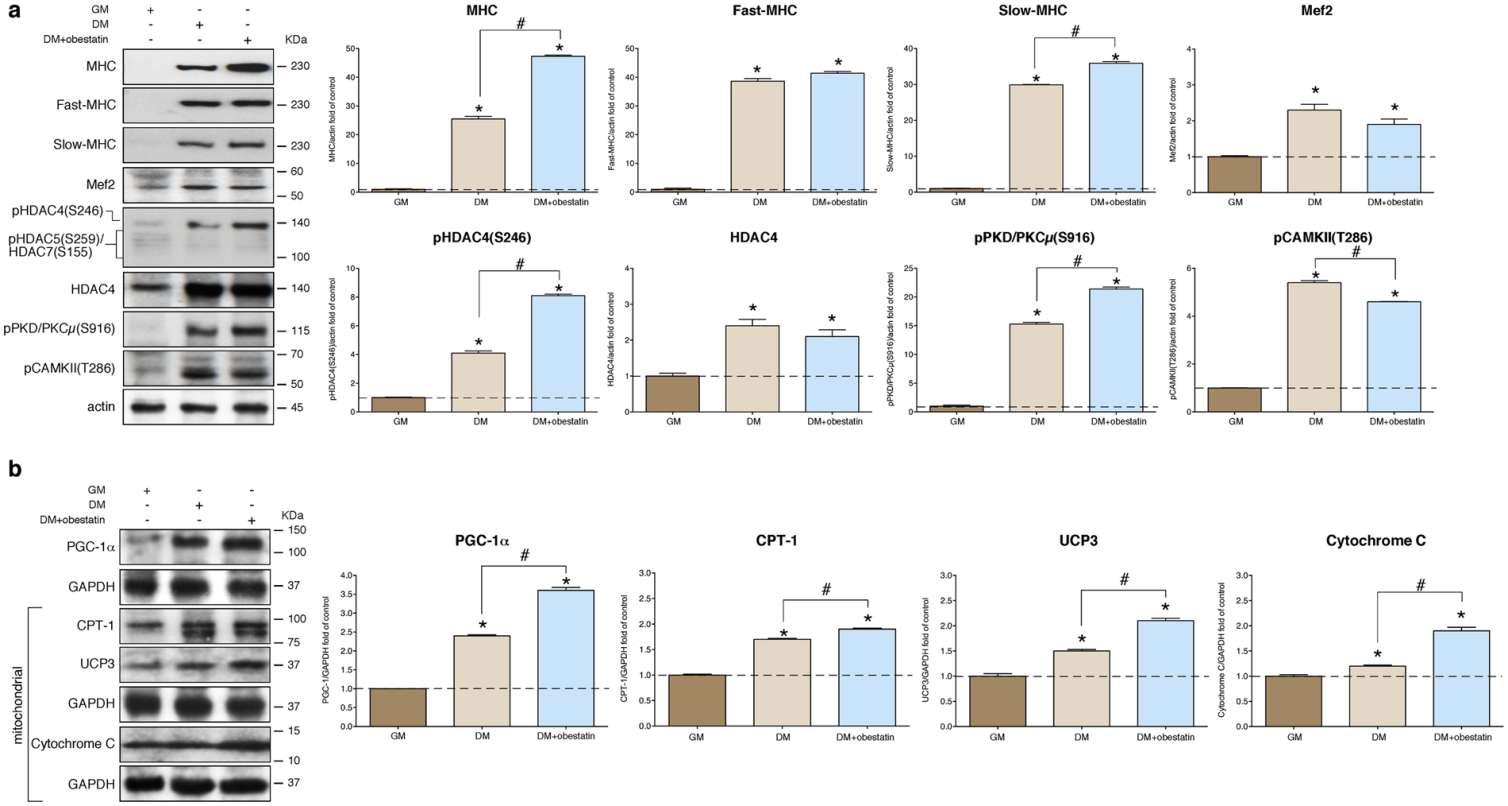
Obestatin signaling regulates the expression of slow contractile protein expression during human myotube formation. (**a**) Western blot analysis of slow-MHC, fast-MHC, Mef2, pHDAC(S246), pHDAC5(S259), pHDAC7(S155), HDAC4, pPKD/PKCµ(S916) and pCAMKII(T286) in human KM155C25 myoblasts (GM) and KM155C25 myotubes at the -day point under DM (control) or DM + obestatin (10 nM). Immunoblots are representative of mean values from each group. (**b**) Western blot analysis of of PGC-1, CPT-1, UCP-3 and Cytochrome C in human KM155C25 myoblasts (GM) and KM155C25 myotubes at the 7-day point under DM (control) or DM + obestatin (10 nM). In **a** and **b**, data show representative blots from one of the three independent experiments. Immunoblots are representative of the mean value. Data were expressed as mean ± SEM obtained from intensity scans (*^,#^*p* < 0.05 *versus* control values).

To investigate whether the obestatin/GPR39 signaling in skeletal muscle is conserved between human and mouse, we used an *in vitro* cell culture model that closely recapitulates the formation and maintenance of human skeletal muscle: the immortalized human muscle stem-cell line from a control individual (KM155C25 Clone 48 cells; for details see Methods**)**. KM155C25 myoblast cells were switched to DM supplemented with obestatin (10 nM) for 7 days^11^. Myoblasts in GM was used as control. As shown in Fig. 8a, the protein levels of total MHC, as detected by immunoblot, was up-regulated in the presence of 10 nM obestatin (~85% increase related to DM cells). Concomitant with this increased MHC expression, stimulation with obestatin stimulation resulted in ~40% increase in the levels of slow-MHC related to untreated control cells, whereas no effect was observed on fast-MHC levels (Fig. 8a). Despite obestatin stimulated slow-MHC levels, no significant differences in Mef2 protein was observed between untreated control cells (DM) and obestatin-treated cells (Fig. 8a). However, levels of pHDAC4(S246) were significantly higher in obestatin-treated cells, representing a 98% increase as compared to untreated control cells (Fig. 8a). Likewise, levels of pPKD/PKCµ(S916) were significantly increased in obestatin-treated cells (40% increase compared to untreated control cells; Fig. 8a). Finally, levels of pCAMKII(T286) were significantly reduced (16% reduction compared to untreated control cells; Fig. 8a). Therefore, HDAC4 activity appears under regulation of PKD to modulate Mef2 target genes in response to obestatin in human cells. Additionally, PGC-1α protein was up-regulated after obestatin stimulation, representing a 50% increase compared to untreated control cells (DM; Fig. 8b). This up-regulation was concomitant with the increased levels in mitochondrial proteins, such as Cytochrome C, CPT-1, and UCP-3 (58, 12 and 40% increase over DM cells; Fig. 8b). Thus, human myotubes underwent functional changes characteristic of oxidative type myotube orchestrated by obestatin signaling during myogenesis.

## Discussion

The capacity of skeletal muscle fibers to remodel relies on regulatory signals that ultimately modify the fiber type by eliciting changes in gene expression. In the present study, we demonstrate that obestatin controls the fiber type determination. This is particularly true concerning the formation of oxidative slow-twitch fibers: (1) in the model of skeletal mouse muscle regeneration after injury, obestatin treatment significantly enhanced muscle regeneration, myofiber hypertrophy and muscle strength; (2) obestatin signaling specifically regulated oxidative slow fiber expression in a coordinated manner involving both class II HDAC/Mef2 and PGC-1α mechanisms linking the activated receptor with distinct sets of accessory and effector proteins, thereby controlling the formation of oxidative muscle fibers; and, (3) obestatin similarly induced a shift in expression in myotube toward slow-twitch during human myogenesis. It is well establish that the obestatin/GPR39 system operates as an autocrine signal in the regulation of skeletal myogenesis^9–11^. Our data add a new component by which this peptide can regulate fiber type in skeletal muscle. This emphasizes the importance of this system in regulating both myogenesis and muscle metabolism.

Obestatin leads to a marked decrease in the number of glycolytic fibers and a significant increase in the number of oxidative fibers during the regeneration process, leading to a muscle with a higher density of oxidative fibers as evidenced by (1) biochemical and immunohistochemical analysis, as well as (2) a leftward shift in the force-frequency curve, characteristic of a shift in fiber types toward an increase in slow-twitch fibers. The decrease in glycolytic fibers in obestatin-treated mice during muscle regeneration is accompanied by an increase of total muscle mass and cross sectional area of muscle fibers, with a broadening of distribution and a shift towards fibers with larger size affecting both oxidative and glycolytic fibers equally. Since obestatin significantly enhances muscle regeneration by simulating satellite stem cell expansion as well as myofiber hypertrophy^10,11^, we believe obestatin exerts a dual action on the regulation of fiber type specification and fiber size in regenerating muscle. A sophisticated signaling-transcription network regulates muscle fiber type. Multiple regulatory factors sense Ca^2+^ (calcineurin and CAMK) and metabolic stress (AMPK and PKD) converging on transcriptional factors (NFAT and Mef2) and repressors (HDACs) regulate slow-twitch muscle gene expression and fiber type transformation^52^. Although AMPK was shown to be a major signaling molecule involved in specifying skeletal muscle fiber type differentiation and mitochondrial biogenesis^53–55^, obestatin does not activate AMPK in mouse skeletal muscle and both human and murine cell lines^9,11^. However, the increase in oxidative fiber proportion occurs together with significant changes in the activation of PKD and CAMK, enzymes known to regulate muscle fiber determination via phosphorylation of class II HDCAs on a series of conserved serine residues. This phosphorylation promotes the nuclear-to-cytoplasmic shuttling of these HDACs and the subsequent activation of Mef2, a transcription factor that promotes the establishment of slow oxidative myofibers^46,48,50,56,57^. Indeed, obestatin increases HDAC phosphorylation, thus providing docking sites for the 14-3-3 chaperone protein leading to nuclear export of HDACs. Signal-dependent nuclear export of class II HDAC proteins allows sustained activation of Mef2-dependent genes, such as slow-twitch contractile protein genes, myoglobin and slow troponin I^50,58,59^. Consistent with the role of Mef2 as a transcriptional regulator of the slow-fiber phenotype, the expression of slow-MHC, myoglobin and slow troponin I was much higher in obestatin-treated muscles than in control muscles. The increase in oxidative fiber density by obestatin/GPR39 activation occurs also with significant changes in several known regulators of mitochondrial function, such as Cytochrome C, UCP-1 and CPT-1. The enhanced mitochondrial capacity was concomitant to increased expression of PGC-1α, a master regulator of mitochondrial gene expression involved in activation of mitochondrial biogenesis and oxidative metabolism^51^. The ability of PKD and CAMK to promote the phosphorylation of class II HDAC, which mediates signal-dependent nuclear export and activation of Mef2, outlines a combinatorial effect of the activation of multiple signaling pathways associated to the obestatin/GPR39 system. Mechanistically, PGC-1α regulates fiber type switching through the coactivation of Mef2^60^. Besides, Mef2 regulates PGC-1α expression through an autoregulatory loop^46,50,61,62^. Thus, Mef2 serves as a nodal point for the control of multiple downstream transcriptional regulators of the slow-fiber phenotype and PGC-1α expression, an effect that might be increased by CAMK signaling pathway^45^. In addition, PGC-1α regulates mitochondrial biogenesis and function in response to obestatin.

As noted above, obestatin leads to a dose-dependent decrease in glycolytic fiber number concomitant to an increase in oxidative fibers after muscle regeneration. However, this loss of glycolytic fibers does not exceed the increase in oxidative fibers and the overall fiber number remains constant. Thus, obestatin determines the molecular regulation of fiber-type determination after muscle injury. Additionally, obestatin leads to a dose-dependent increase in glycolytic and oxidative fiber size. Concomitant increases in size and oxidative capacity appears to be limited by an interaction between intracellular signaling pathways. In fact, the lack of AMPK signaling under obestatin stimulation might intensify growth signaling by activating the major signaling network for protein synthesis, the Akt/mTOR pathway^63–65^. It was shown that AMPKα1/α2-knockout mice possess fibers with greater cross sectional area^66^, supporting the notion that AMPK negatively influences muscle growth. However, despite AMPK mediates metabolic adaptation by enhancing activity and expression of PGC-1α, which coordinates expression of nuclear- and mitochondrial-encoded genes critical for mitochondrial biogenesis and increased oxidative capacity^67^, the obestatin effect supports the notion that hypertrophy and enhanced oxidative capacity can occur simultaneously in skeletal muscle and demonstrate that the signaling mechanisms controlling both events are independently regulated.

Our previous studies have established that the obestatin/GPR39 system induces productive repair and growth by stimulating both satellite cell expansion and myofiber hypertrophy. The results from the present work demonstrate that this peptide not only increases muscle mass, but also leads to increased force and a shift in fiber type toward slow-twitch fibers. Taken together, these findings support a potential use of obestatin as a therapeutic target for skeletal muscle myopathies related to muscle regeneration, since enhanced oxidative capacity affords protection against contraction-induced damage in dystrophic muscles^68,69^. However, any proposition of therapeutic strategies will require extensive studies of the obestatin/GPR39 signaling in a human context. Although murine models are widely used to identify and test drug candidates, the assumption that murine models translate directly to human conditions has been challenged in different studies^70–74^. Obestatin treatment similarly induced a shift in fiber type toward slow-twitch during human myogenesis *in vitro*, involving also class II HDAC phosphorylation and PGC-1α up-regulation linked to significant changes in regulators of mitochondrial function. However, unlike in mice, the increase in human oxidative myotube density after obestatin treatment occurs with no significant changes in Mef2 expression or CAMK activation. These signaling differences may be attributed to differences in signaling kinetics of effector proteins such as CAMK between humans and mice^11^. Despite these differences, the overall effect of the obestatin/GPR39 signaling on the development of oxidative myotubes is conserved between humans and mice.

In conclusion, obestatin signaling plays a role in the formation, contractile properties and metabolism of skeletal muscle through the determination of oxidative fiber type. Our data indicate that this peptide regulates Mef2 activity and PGC-1α expression. Both mechanisms result in a shift in muscle metabolism and function. The increase in Mef2 and PGC-1α signaling activates oxidative capacity, whereas Akt/mTOR signaling positively regulates myofiber growth. Thus, obestatin has an important role in the regulation of oxidative muscle development.

## Experimental Procedures

### Materials

Rat/mouse obestatin was obtained from Barcelona Peptides S.A. (Barcelona, ES). Human obestatin was obtained from California Peptide Research (Napa, CA, USA). Antibodies used are listed in Table S1 (supplementary information). All other chemical reagents were from Sigma Chemical Co. (St. Louis, MO, US).

### Animal care

This study used 10-weeks-old male Swiss mice (40 g) obtained from Charles Rivers Laboratories. Mice were housed in 12 hours light/12 hours dark cycles with free access to standard mice chow diet and water. All experimental procedures were approved by the Animal Care Committee of the Universitat Autònoma de Barcelona (UAB; Procedure number 3048; Barcelona, ES) according to the guidelines of the Spanish Royal Decree 53/2013, Directive 2010/63/EU and FELASA Guidelines.

### Freeze-induced muscle injury and obestatin dosing

The experimental protocol used in the present work was previously described^10^. Under anesthesia, the right hindlimb of the mice was shaved and the tibialis anterior muscles (TA) were exposed via a 1-cm-long incision in aseptically prepared skin overlying the muscle. Traumatic freeze injury was induced by applying a 120-mm-diameter steel probe, pre-cooled to the temperature of dry ice (−79 °C), to the belly of the TA muscle for 10 seconds. After injury procedure, the skin incision was closed using 6–0 silk sutures. This procedure induced a focal injury extending distally from the spike of the tibia and spreading over approximately one-third of the muscle. The average length and maximal cross-sectional area of the lesion sites, evaluated by Evans Blue labeling^10^, were 3002 ± 12 µm and 3675690 ± 27501 µm^2^, respectively (mean ± SEM). Contralateral hindlimb was used as uninjured control. Animals were assigned to one of the following experimental groups (n = 5 per group): (1) normal control group (no muscle injury, no treatment); (2) muscle injury group (muscle injury, no treatment); (3) muscle injury group+vehicle (muscle injury, vehicle administration); and (4) obestatin-treated group (muscle injury, obestatin administration). Obestatin treatment was performed via injection of 20 µL of obestatin solution in PBS (pH 6.3) [100, 300 or 500 nmol/kg body weight] into the target muscles at defined time points during 30 days. Muscle injury group+vehicle was treated with 20 µL of PBS under the same conditions. Injections were performed twice per muscle inserting the needle of a 0.3 mL/29 gauge syringe (Terumo Myjector V-100 insulin syringe) at 1 mm in the distal part of the muscle, in a region that is not taken into account for histological analysis, as previously described^10^. After 30-days, TA muscles from different groups were excised and processed for immunoblot and immunohistochemical analyses.

### Force measurement

Mice were anaesthetized with isofluorane and put on a heating pad to maintain body and muscles at 37 °C. The distal tendon of the TA muscle was attached to a Pioden control LTD Dynamometer UF1 force transducer, which was connected to a Lectromed MT8P physiograph (Letchworth, Herts, UK). An output of the polygraph was also connected to a digital data acquisition system (PowerLab/800 AD-Instruments, Castle Hill, NSW, AUT) to acquire force at a sampling rate of 5 kHz. TA was kept moist with a physiological solution containing 118.5 mM NaCl, 4.7 mM KCl, 1.3 mM CaCl_2_, 3.1 mM MgCl_2_, 25 mM NaHCO_3_, 2 mM NaH_2_PO_4_, and 5.5 mM D-glucose continuously gassed with a mixture of O_2_:CO_2_ (95:5) to maintain a pH = 7.4. Contractions were evoked every 100 seconds (s) by field stimulation using one electrode placed on a short section of the sciatic nerve. The electrode was connected to an electric stimulator (SRI, Scientific and Research Instruments Ltd, UK) and single twitches were elicited with one 0.3 ms square pulse at 10 V. Maximum force was measured during a tetanic contraction with 200 ms train of pulses at 100 or 200 Hz.

### Cell culture and differentiation

Mouse C2C12 myoblasts were cultured as described by the supplier (ECACC, Whiltshire, UK). Briefly, C2C12 myoblasts were maintained in growth medium (GM) containing DMEM supplemented with 10% fetal bovine serum (FBS), 100 U/mL penicillin, and 100 U/mL streptomycin. For differentiation, cells were expanded to 80% confluence and GM was replaced with differentiation medium (DM; DMEM supplemented with 2% FBS, 100 U/mL penicillin, and 100 U/mL streptomycin) for 7 days unless otherwise stated. The immortalized human myoblast cell line, KM155C25 Clone 48, was obtained from the platform for immortalization of the Myology Institute (Paris, France). It was immortalized from a biopsy obtained with an official consent through MYOBANK-BTR (Bank of Tissues for Research, a partner in the EU network EuroBioBank; gracilis muscle, donor age 25 years)^75^. KM155C25 Clone 48 cells were cultivated in growth medium (GM) containing Medium 199:DMEM (1:4, v-v; Lonza, Pontevedra, SP) supplemented with 20% FBS (v/v), 25 µg/µL fetuin, 5 ng/mL hEGF, 0.5 ng/mL bFGF, 0.2 µg/mL dexamethasone (Sigma-Aldrich; MO, USA) and 50 µg/mL gentamycin (Invitrogen) as described previously^11^. Differentiation into myotubes was initiated at 90% confluence by switching to differentiation medium [DM; DMEM supplemented with 50 µg/mL gentamycin (Invitrogen) and either 10 µg/mL insulin or 10 nM obestatin] for 7 days unless otherwise stated.

### Histology and immunofluorescence

Muscle samples were mounted in tissue freezing medium (gum tragacanth) and snap frozen in nitrogen-cooled isopentane. The sections, 10 µm thick, were mounted on Histobond Adhesion Microslides (Marienfeld, Lauda-Königshofen, DE). For the haematoxylin/eosin (HE) and succinate dehydrogenase (SDH) staining’s, serial cryostat sections were stained following a standard protocol. Immunofluorescence analysis of MHC expression was performed with primary antibodies against MHCI, MHCIIa, MHCIIb and MHCIIx following the protocol previously described^76^. Primary and secondary antibodies used are listed in Table S1 (supplementary information). For quantification of myofiber cross-section area, the cryostat sections were stained with HE and SDH and five randomly chosen microscopic fields from two different sections in each tissue block were examined using ImageJ64 analysis software. For fiber type analysis, all fibers within the entire muscle/cross-section were characterized.

### Immunoblot analysis

The tissue or the cell samples were directly lysed in ice-cold RIPA buffer [50 mM Tris-HCl (pH 7.2), 150 mM NaCl, 1 mM EDTA, 1% (v/v) NP-40, 0.25% (w/v) Na-deoxycholate, protease inhibitor cocktail (Sigma Chemical Co, St. Louis, MO, US), phosphatase inhibitor cocktail (Sigma Chemical Co, St. Louis, MO, US)]. The lysates were clarified by centrifugation (14,000 × g, 15 min at 4 °C) and the protein concentration was quantified using the QuantiPro^TM^ BCA assay kit (Sigma Chemical Co, St. Louis, MO, US). For immunoblotting, equal amounts of protein were fractionated by SDS-PAGE and transferred onto nitrocellulose membranes. Immunoreactive bands were detected by enhanced chemiluminescence (Pierce ECL Western Blotting Substrate; Thermo Fisher Scientific, Pierce, Rockford, IL, US).

### Data analysis

All values are presented as mean ± standard error of the mean (SEM). T-tests were carried out for comparisons between two samples. Unpaired T-test was used to assess the statistical significance of one-way or two-way analysis when the test statistic followed a normal distribution. For multiple comparisons, ANOVA was employed. *p* < 0.05 was considered as statistically significant (*, #, &, $).

## Electronic supplementary material

Supplementary information
