# Publisher Correction: Obestatin controls skeletal muscle fiber-type determination

**DOI:** 10.1038/s41598-018-36660-1

**Published:** 2018-12-14

**Authors:** Icía Santos-Zas, Tania Cid-Díaz, Jessica González-Sánchez, Uxía Gurriarán-Rodriguez, Carlos Seoane-Mosteiro, Begoña Porteiro, Rubén Nogueiras, Xesús Casabiell, José Luis Relova, Rosalía Gallego, Vincent Mouly, Yolanda Pazos, Jesus P. Camiña

**Affiliations:** 1 Área de Endocrinología Celular y Molecular, Instituto de Investigación Sanitaria de Santiago (IDIS), Complejo Hospitalario Universitario de Santiago (CHUS), Servicio Gallego de Salud (SERGAS), Santiago de Compostela, Spain; 2 Sprott Center for Stem Cell Research, Ottawa Hospital Research Institute, Ottawa, ON K1H8L6 Canada; 3 Departamento de Fisiología, Universidad de Santiago de Compostela (USC), Santiago de Compostela, Spain; 4 Departamento de Ciencias Morfológicas, USC, Santiago de Compostela, Spain; 5 Sorbonne Universités, UPMC Univ Paris 06, INSERM UMRS974, CNRS FRE3617, Center for Research in Myology, Paris, France

Correction to: *Scientific Reports* 10.1038/s41598-017-02337-4, published online 18 May 2017

In the original version of this Article, the author José Luis Relova was incorrectly indexed. This error has now been corrected.

