# Supplementary material for: Obestatin controls skeletal muscle fiber-type determination
Source: Sci Rep. 2017 May 18;7:2137. doi: 10.1038/s41598-017-02337-4 (PMC5437042; doi:10.1038/s41598-017-02337-4)

**Obestatin controls skeletal muscle fiber-type determination**

Icía Santos-Zas^1^, Tania Cid-Díaz^1^, Jessica González-Sánchez^1^, Uxía Gurriarán-Rodriguez^2^, Carlos Seoane-Mosteiro^1^, Begoña Porteiro^3^, Rubén Nogueiras^3^, Xesús Casabiell^3^, José Luis Relova^3^, Rosalía Gallego^4^, Vincent Mouly^5^, Yolanda Pazos^1^, Jesus P Camiña^1,*^

^1^Área de Endocrinología Celular y Molecular, Instituto de Investigación Sanitaria de Santiago (IDIS), Complejo Hospitalario Universitario de Santiago (CHUS), Servicio Gallego de Salud (SERGAS), Santiago de Compostela, Spain.

^2^Sprott Center for Stem Cell Research, Ottawa Hospital Research Institute, Ottawa, ON K1H8L6, Canada.

^3^Departamento de Fisiología, Universidad de Santiago de Compostela (USC), Santiago de Compostela, Spain.

^4^Departamento de Ciencias Morfológicas, USC, Santiago de Compostela, Spain.

^5^Sorbonne Universités, UPMC Univ Paris 06, INSERM UMRS974, CNRS FRE3617, Center for Research in Myology, Paris, France.

Keywords: skeletal muscle | skeletal muscle regeneration | obestatin signaling | muscle force | fiber-type determination

Short title: Obestatin and fiber-type determination

**Table S1**

| **Antibody** | **Use** | **Dilution/**  **concentration** | **Supplier** | **Reference** |
| --- | --- | --- | --- | --- |
| Myosin heavy chain | WB* | 1:1000 | Hibridoma BanK | MF20 |
| Myosin heavy chain I | IF** | 2 µg/mL | Hibridoma BanK | BA-F8 |
| Myosin heavy chain IIa | IF | 2 µg/mL | Hibridoma BanK | SC-71 |
| Myosin heavy chain IIb | IF | 2 µg/mL | Hibridoma BanK | BF-F3 |
| Myosin heavy chain IIx | IF | 2 µg/mL | Hibridoma BanK | 6H1 |
| Goat Anti-Mouse Alexa Fluor 350 IgG2b | IF | 1:1000 | Life Technologies | A-21140 |
| Goat Anti-Mouse Alexa Fluor 594 IgG1 | IF | 1:1000 | Life Technologies | A-21125 |
| Goat Anti-Mouse Alexa Fluor 488 IgM | IF | 1:1000 | Life Technologies | A-10680 |
| Slow myosin heavy chain | WB | 1:1000 | Sigma | M8421 |
| Fast myosin heavy chain | WB | 1:2000 | Sigma | M4276 |
| Actin | WB | 1:5000 | Abcam | Ab1801 |
| Myoglobin | WB | 1:1000 | BioGenex | AM012 |
| Troponin I-SS | WB | 1:1000 | Santa Cruz | Sc20645 |
| GAPDH | WB | 1:1000 | Abcam | Ab9485 |
| HDAC4 | WB | 1:1000 | Cell Signaling | 7628 |
| pCAMKII(T286) | WB | 1:1000 | Cell Signaling | 3361 |
| pHDAC4 (Ser246)/ pHDAC5 (Ser259)/ pHDAC7 (Ser155) | WB | 1:1000 | Cell Signaling | 3443 |
| pPKD (Ser744/748) | WB | 1:1000 | Cell Signaling | 2054 |
| Mef2 | WB | 1:1000 | Cell Signaling | 9736 |
| PGC-1α | WB | 1:1000 | Santa Cruz | Sc13067 |
| CPT-1 | WB | 1:1000 | Santa Cruz | Sc139480 |
| UCP3 | WB | 1:1000 | Santa Cruz | Sc31387 |
| Cytochrome C | WB | 1:1000 | Santa Cruz | Sc7159 |

*WB: western blot; **IF: Immunofluorescence


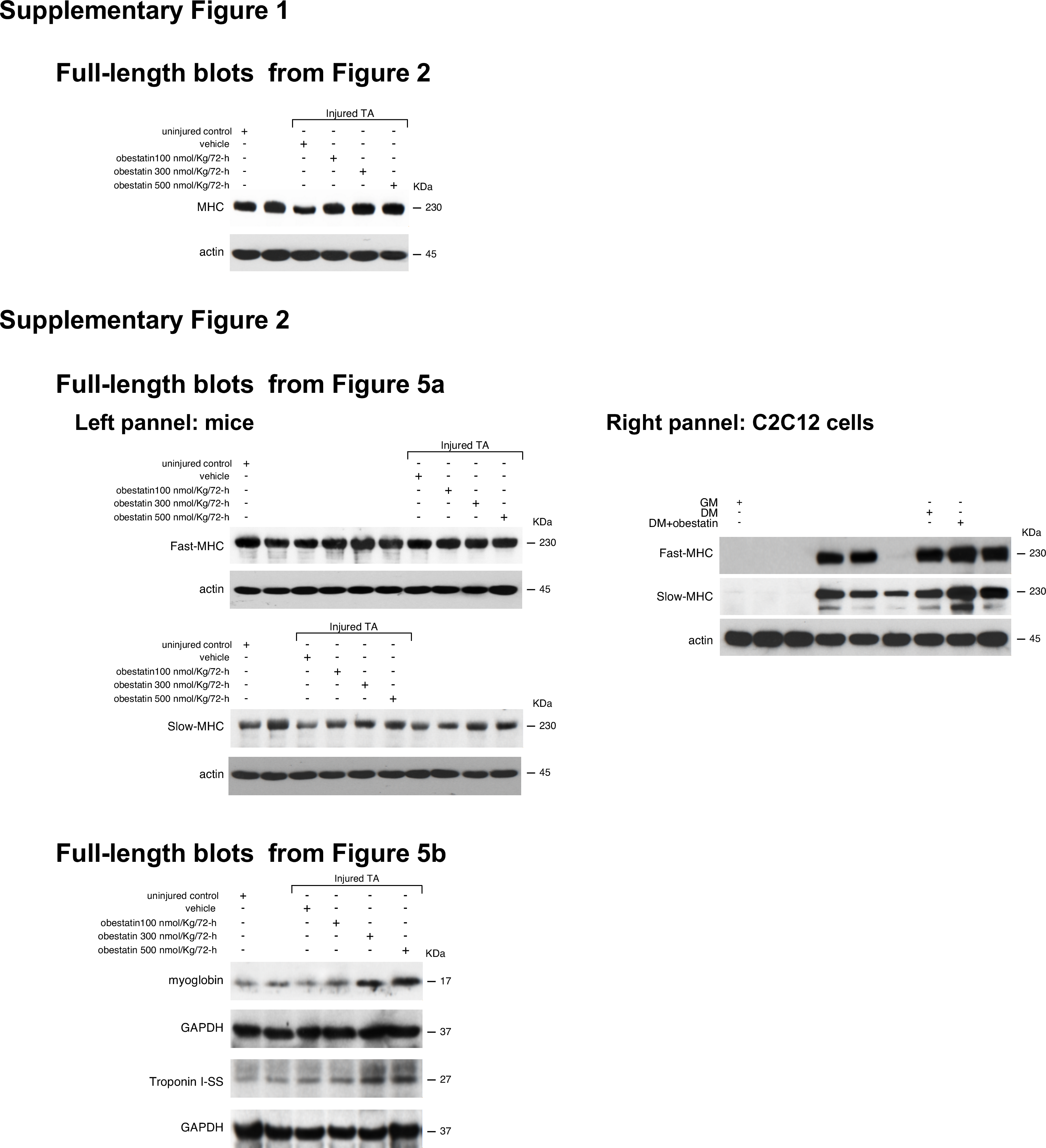


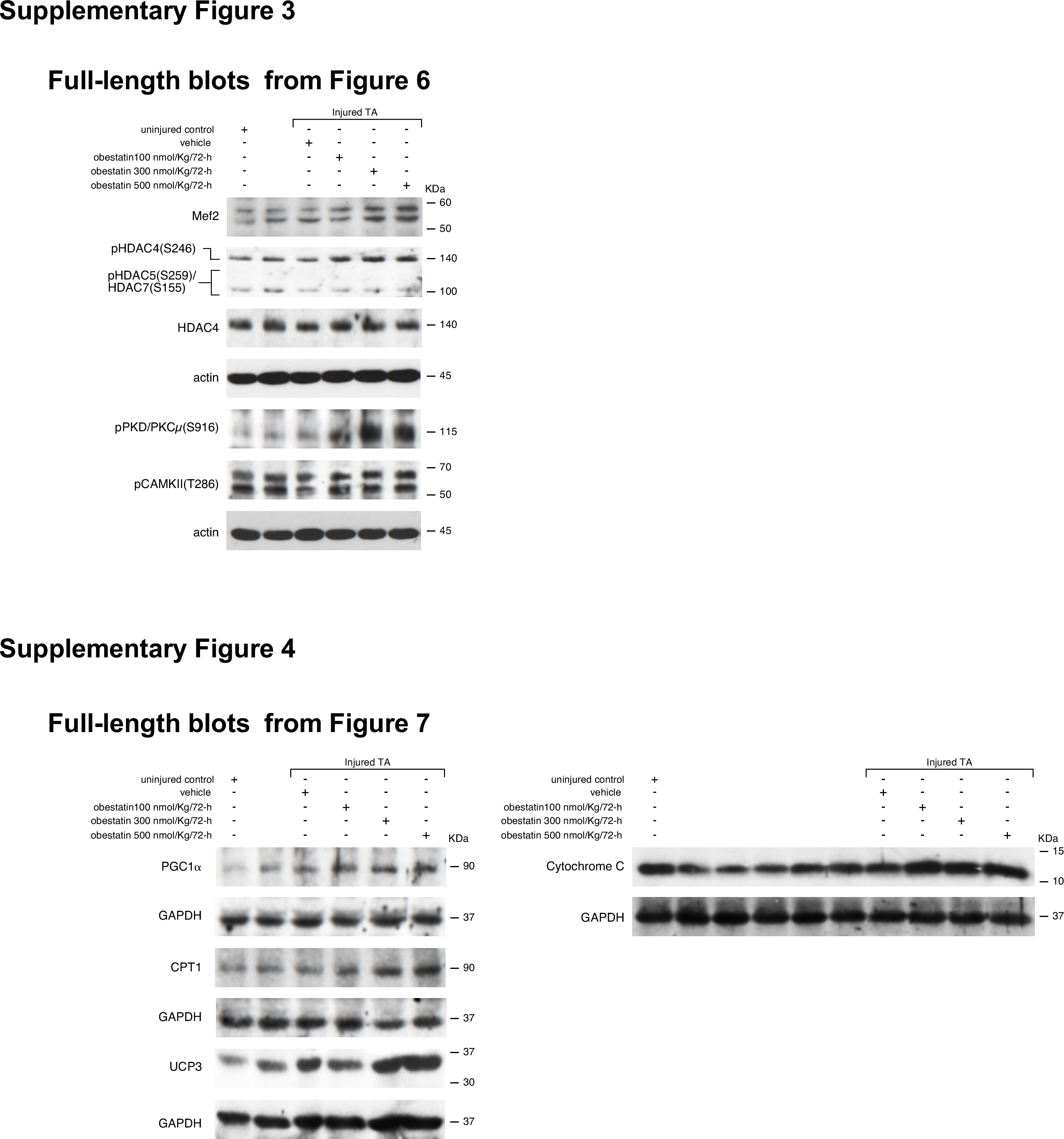

Supplement: Supplementary file 1 — Supplementary information [file 41598_2017_2337_MOESM1_ESM.doc]
